# HLA-Modeler: Automated Homology Modeling of Human Leukocyte Antigens

**DOI:** 10.1155/2013/690513

**Published:** 2013-11-27

**Authors:** Shinji Amari, Ryoichi Kataoka, Takashi Ikegami, Noriaki Hirayama

**Affiliations:** ^1^Science and Technology Systems Division, Computational Science Department, Ryoka Systems Inc., 1-1-2 Oshiage, Sumida-ku, Tokyo 131-0045, Japan; ^2^Basic Medical Science and Molecular Medicine, Tokai University School of Medicine, 147 Shimokasuya, Isehara, Kanagawa 259-1143, Japan

## Abstract

The three-dimensional (3D) structures of human leukocyte antigen (HLA) molecules are indispensable for the studies on the functions at molecular level. We have developed a homology modeling system named HLA-modeler specialized in the HLA molecules. Segment matching algorithm is employed for modeling and the optimization of the model is carried out by use of the PFROSST force field considering the implicit solvent model. In order to efficiently construct the homology models, HLA-modeler uses a local database of the 3D structures of HLA molecules. The structure of the antigenic peptide-binding site is important for the function and the 3D structure is highly conserved between various alleles. HLA-modeler optimizes the use of this structural motif. The leave-one-out cross-validation using the crystal structures of class I and class II HLA molecules has demonstrated that the rmsds of nonhydrogen atoms of the sites between homology models and crystal structures are less than 1.0 Å in most cases. The results have indicated that the 3D structures of the antigenic peptide-binding sites can be reproduced by HLA-modeler at the level almost corresponding to the crystal structures.

## 1. Introduction

The cause of various diseases involves the human leukocyte antigen (HLA) system which is the human version of the major histocompatibility complex. The HLA genes involved in the immune response fall into two classes, I and II, which are structurally and functionally different. Typical diseases associated with HLA molecules are autoimmune diseases [1] and infectious diseases [2]. The association between specific HLA alleles and adverse drug reactions which frequently cause significant morbidity and mortality for patients is also widely known [3]. A reliable three-dimensional (3D) structure of the particular HLA allele responsible for the specific event is essential to understand the underlying molecular mechanism in order to develop effective therapeutic agents or/and countermeasures. Since the pioneering work by Wiley et al. [4], various crystal structures of the HLA molecules have been disclosed so far. The crystal structures have shown that the peptide-binding groove of an HLA molecule consists of two parts, a floor and two walls. Although this canonical topology is highly conserved among different alleles, certain structural differences exist depending on the alleles. Therefore, the 3D structure of a particular HLA molecule is required for HLA studies. The HLA genes are the most polymorphic in the human genome and there are a large number of allelic variations. In the case of alleles belonging to the isotype A of class I HLA, the number of independent alleles deposited in the IMGT/HLA database [5] is 1,372 as of 27 July 2013 (version 3.13.1). On the other hand, the number of the corresponding nonredundant alleles whose 3D structures are deposited in Protein Data Bank [6] as of 24 April 2013 is just nine. This shows that a huge gap exists between known allele sequences and available 3D structures. Therefore, it is reasonable to assume that experimental structures for all the possible HLA alleles will not be available in the near future.

In the absence of experimental structures, *in silico *homology modeling can provide a viable alternative to generate reasonably accurate models of the allele of interest. Homology modeling is a methodology to predict protein structure based on the general observation that proteins with similar sequences have similar structures. The accuracy of homology models compared to the actual experimental structure is generally judged by C*α* atomic pair root-mean-square deviation (rmsd). Depending on the degree of sequence identity or similarity and the quality of the alignment, the rmsd can be up to ca. 1-2 Å [7].

Homology modeling package specialized in HLA molecules is not available until now as far as we know. The purpose of this study is to create an automated HLA modeling application suitable for HLA studies at the molecular level.

## 2. Methods

**Figure 1:**
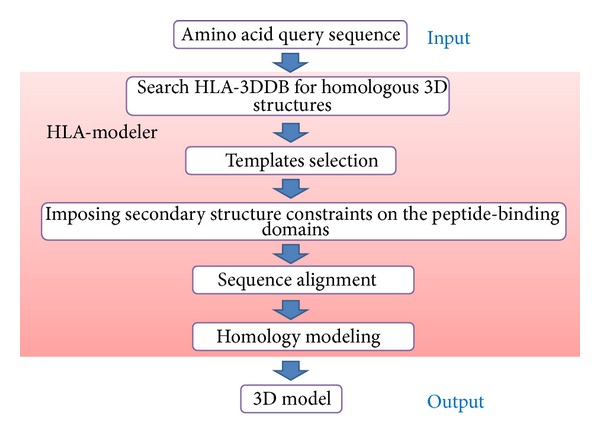
A flow chart of HLA-modeler.

The application named HLA-modeler was coded by the use of scientific vector language (svl) implemented in MOE [8]. All of the crystal structures of the HLA molecules deposited in the PDB were retrieved and a local HLA structural database named HLA-3DDB was compiled on April 24th, 2013. Information items stored in HLA-3DDB are given in Table 1. The data forthe PDB entry of 1AO7 are given as examples. The proper allele names were obtained from IMGT/HLA. The template structure which is most homologous to the query sequence is selected from HLA-3DDB and used for homology modeling. Therefore, the only required input data is a query sequence. Segment matching algorithm [9] implemented in MOE is used for homology modeling. The optimization of the models is carried out by use of the PFROSST force field [10, 11] considering the implicit solvent model [12]. Multiple intermediate structures are constructed. The best structure in terms of the free energy of hydration calculated based on generalized Born/volume integral implicit solvent model [12] is selected. In the final optimization of the structure, nonhydrogen atoms are tethered. The antigenic peptide-binding site is primarily used in HLA-modeler in order to best use the 3D characteristics of the essential site of the template structure. A flow chart of HLA-modeler is shown in Figure 1.

Specific binding of antigenic peptides to a particular HLA molecule is a central problem for most of the HLA studies. HLA-modeler can construct the homology model of HLA-peptide complex based on the supplied peptide sequence. The svl code of HLA-modeler is available from Ryoka Systems Inc. on request.

## 3. Results and Discussion

### 3.1. Validation of Homology Models Constructed by HLA-Modeler

**Figure 2:**
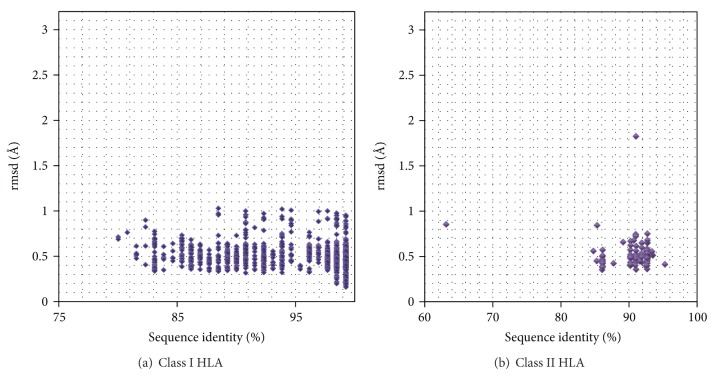
Rmsds of nonhydrogen atoms of antigenic peptide-binding sites between the crystal structures and the homology models.

It is of interest to validate the reproducibility of homology models routinely built by HLA-modeler. For this purpose, the leave-one-out cross-validation was undertaken. In the current HLA-3DDB, there are 41 and 27 crystal structures of nonredundant HLA molecules belonging to classes I and II, respectively. For each amino acid sequence in HLA-3DDB, homology models were constructed using all structures belonging to the same class except the identical structure as template structures. The results for classes I and II are illustrated in Figures 2(a) and 2(b), respectively. The rmsds of nonhydrogen atoms in the peptide-binding site are shown. The rmsds do not depend on the sequence identity and are generally lesser than 1.1 Å. The only exception is the structure of HLA-DR1 (PDB ID: 4GBX) modeled based on the template structure of HLA-DR52c (3C5J). Since the structure of 4GBX is the heterodimer between HLA-DR1 and HLA-DM, the structure of the HLA-DR1 molecule is deformed due to the interaction with HLA-DM. This is the major reason of the large rmsd of ca. 1.8 Å. If all domains are used for homology modeling, rmsds are up to 3.0 and 2.7 Å in classes I and II, respectively. It indicates that the homology modeling strategy adopted in HLA-modeler is suitable to construct the structure of antigenic peptide-binding site.

### 3.2. Two Examples of Homology Modeling of Class I HLA Molecules

**Figure 3:**
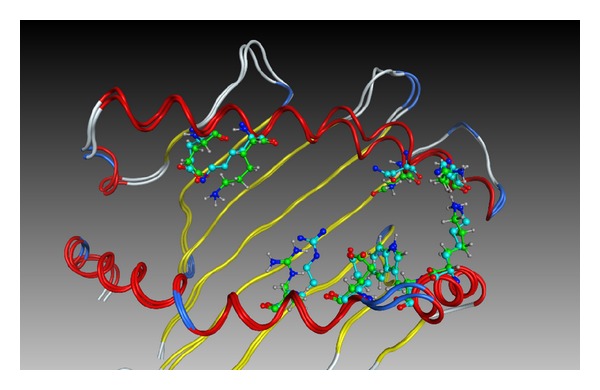
Superposition of nonhydrogen atoms of peptide-binding sites of the crystal structure and the homology model of HLA-C∗04:01. The residues whose positions are significantly different are depicted by ball-and-stick model. Green and blue colors of the carbon atoms denote the homology model and the crystal structure, respectively.

**Figure 4:**
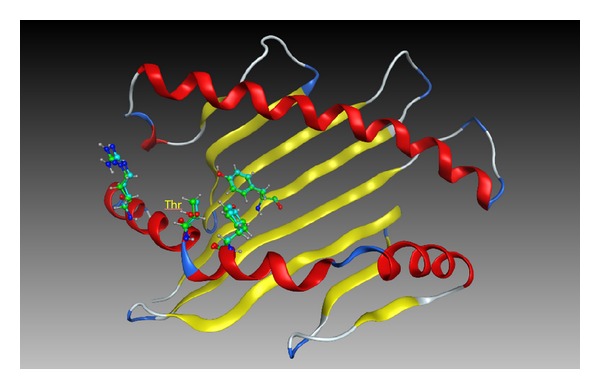
Superposition of the nonhydrogen atoms of the peptide-binding sites of the crystal structure and the homology model of HLA-A∗02:01. The mutated amino acid of Thr is labelled. Three residues whose positions are significantly different are shown by ball-and-stick model. Green and blue colors of the carbon atoms denote the homology model and the crystal structure, respectively.

In the first case, the identity between the query and the template sequences is 78.4%. This sequence identity is significantly low among HLA molecules belonging to the same class. A homology model was built using the sequence of the HLA-C∗04:01 molecule (PDB ID: 1QQD) as a query sequence, and the structure of the HLA-B∗44:02 molecule (PDB ID: 1M60) is selected as a template structure. The nonhydrogen atoms of the *α*1 and *α*2 domains of the homology model and the crystal structure are superimposed in Figure 3. The rmsd is 0.7 Å. The amino acid residues whose positions differ significantly between two structures are depicted. It is considered that such degree of discrepancy as shown in Figure 3 may be small enough for most qualitative analysis such as estimation of amino acid residues which should possibly bind to antigenic peptides. Even in the cases where sequence identity is low, reasonably accurate models can be constructed as illustrated in Figure 2. However, if it is necessary to predict the conformations of amino acid residues at the peptide-binding site as accurate as possible, it is better to use the template structure with higher sequence identity. In the second case of homology modeling of the HLA-A∗02:01 molecule (PDB ID: 1LP9) based on the template structure of a mutant molecule of HLA-A∗02:01 (2UWE), the sequence identity is very high (99.2%). Only one residue at the antigenic peptide-binding site is different, that is, Ala and Thr in 2UWE and 1LP9, respectively. The rmsd of nonhydrogen atoms of the peptide-binding sites is 0.23 Å. The superimposed structures are shown in Figure 4. The position of the relevant Thr residue in the homology model is almost identical to that in the crystal structure.

In summary, we can construct reasonably reliable 3D structures of class I HLA molecules by HLA-modeler.

### 3.3. An Example of Homology Modeling of Class II HLA Molecule

Japanese cedar pollinosis is a type I allergic disease caused by Japanese cedar pollen. Hori et al.found that the disease is significantly associated with HLA-DP5 and identified an immunodominant peptide [13]. The minimum antigenic sequence of KVTVAFNQF was suggested. Understanding the interactions between the peptide and the HLA molecule at the molecular level will greatly help to find therapeutic strategies against this disease. However, the 3D structure has not been disclosed so far. By use of HLA-Modeler, we have built the homology model of the HLA-DP5 molecule with the minimum immunodominant peptide.

**Figure 5:**
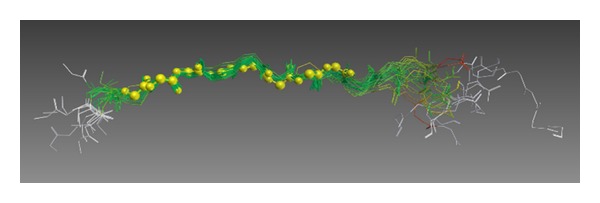
Superposition of nonhydrogen atoms of 28 different antigenic peptides. The yellow spheres indicate the main chain atoms of eight residues in an antigenic peptide. The conformations of these eight residues are conserved among 28 different peptides.

The main chains of the antigenic peptides bound to the HLA molecules take highly conserved extended structures. In particular, the main chain structure of eight residues shown in Figure 5 is conserved. If the main chain atoms of the corresponding eight residues in 28 independent peptides bound to the class II HLA molecules in the crystal structures are superimposed, the median rmsd for each residue ranges from 0.26 to 0.50Å. It indicates that the main chain conformations of the peptides bound to class II HLA are conserved in this particular region. The eight residues involving P1, P4, and P6 anchoring residues should play significant role in binding to the class II HLA molecules.

**Figure 6:**
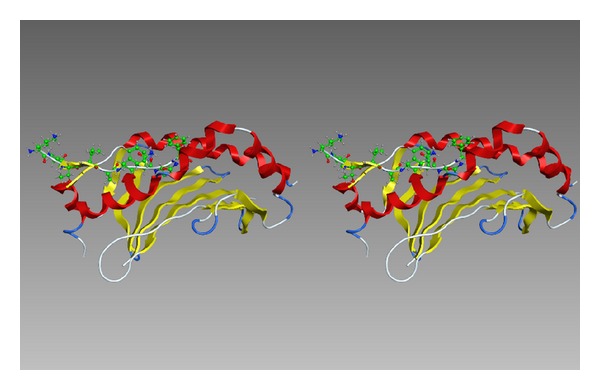
A homology model of HLA-DP5 with the minimum antigenic peptide. The peptide is depicted by ball-and-stick model with the carbon atoms colored in green. The left end is the lysine residue. This picture is a cross-eyed stereo diagram.

By taking the structural conservation of the bound peptides into account, the most plausible binding site of the immunodominant nonapeptide was searched. The nonapeptide was shifted along the template peptide with the sequence of RKFHYLPFLPSTGGS. The structures of the complexes between the HLA DP5 molecule and the nonapeptide with seven different alignments were optimized. The binding affinity of each complex was judged by a scoring function of GBVI/WSA_dG which is considered to express protein-ligand binding free energy [14]. The complex structure with the minimum GBVI/WSA_dG value is shown in Figure 6. The two terminal K and F residues of the peptide protrude from the antigenic peptide-binding groove and point toward the T-cell receptor. The experimental data have demonstrated that these residues are essential for the interactions with T-cell receptor. Therefore, the credibility of this homology model is considered to be high.

## 4. Conclusions

Since there are a large number of allelic variations, it is expected that the 3D structures of all the possible HLA alleles will not be experimentally determined in the near future. Under these circumstances, it is indispensable to use *in silico* methodology to predict the missing structures to promote HLA studies. The present study has demonstrated that if we properly use a strategy to build up homology models based on the structurally conserved antigenic peptide-binding site, we can build the reliable 3D structure of the site which is essential for the functions. The 3D structures of particular HLA molecules are useful to deepen our understanding of the molecular interactions between the HLA molecules and specific antigenic peptides. The 3D models of HLA molecules are also essential to disclose the molecular mechanisms of adverse drugs reactions closely related to specific HLA molecules. Moreover, the 3D models of the HLA molecules associated with certain autoimmune diseases will contribute to the discovery of drugs which could suppress the autoimmune reactions. The automatic modeling system such as HLA-modeler will be indispensable for extensive studies on these topics.

## Figures and Tables

**Table 1:** The information items contained in HLA-3DDB and example data for 1AO7.

Items	Example data (1AO7)
Allele name	A∗02:01
Complex	HLA/peptide/TCR
PDB ID	1AO7
PDB header	Complex between human T-cell receptor, viral peptide (Tax), and HLA-A 0201
Atomic parameters	(a specific format is used in HLA-modeler, but it can be converted into the PDB format)
Date of deposition	1997/7/21
Date of the last modification	2009/2/24
Experiment type	X-ray
Resolution (Å)	2.60
Free *R* value	0.32
Mean *B* value	42.4
Other chemical components	Ethyl mercury ion
